# Informatics assessment of COVID-19 data collection: an analysis of UK Biobank questionnaire data

**DOI:** 10.1186/s12911-024-02743-5

**Published:** 2024-10-31

**Authors:** Craig S. Mayer

**Affiliations:** Lister Hill National Center for Biomedical Communications, National Library of Medicine, NIH, 8600 Rockville Pike, Bethesda, MD 20894 USA

**Keywords:** COVID-19, Informatics, UK Biobank, Survey Questionnaires

## Abstract

**Background:**

There have been many efforts to expand existing data collection initiatives to include COVID-19 related data. One program that expanded is UK Biobank, a large-scale research and biomedical data collection resource that added several COVID-19 related data fields including questionnaires (exposures and symptoms), viral testing, and serological data. This study aimed to analyze this COVID-19 data to understand how COVID-19 data was collected and how it can be used to attribute COVID-19 and analyze differences in cohorts and time periods.

**Methods:**

A cohort of COVID-19 infected individuals was defined from the UK Biobank population using viral testing, diagnosis, and self-reported data. Changes over time, from March 2020 to October 2021, in total case counts and changes in case counts by identification source (diagnosis from EHR, measurement from viral testing and self-reported from questionnaire) were also analyzed. For the questionnaires, an analysis of the structure and dynamics of the questionnaires was done which included the amount and type of questions asked, how often and how many individuals answered the questions and what responses were given. In addition, the amount of individuals who provided responses regarding different time segments covered by the questionnaire was calculated along with how often responses changed. The analysis included changes in population level responses over time. The analyses were repeated for COVID and non-COVID individuals and compared responses.

**Results:**

There were 62 042 distinct participants who had COVID-19, with 49 120 identified through diagnosis, 30 553 identified through viral testing and 934 identified through self-reporting, with many identified in multiple methods. This included vast changes in overall cases and distribution of case data source over time. 6 899 of 9 952 participants completing the exposure questionnaire responded regarding every time period covered by the questionnaire including large changes in response over time. The most common change came for employment situation, which was changed by 74.78% of individuals from the first to last time of asking. On a population level, there were changes as face mask usage increased each successive time period. There were decreases in nearly every COVID-19 symptom from the first to the second questionnaire. When comparing COVID to non-COVID participants, COVID participants were more commonly keyworkers (COVID: 33.76%, non-COVID: 15.00%) and more often lived with young people attending school (61.70%, 45.32%).

**Conclusion:**

To develop a robust cohort of COVID-19 participants from the UK Biobank population, multiple types of data were needed. The differences based on time and exposures show the important of comprehensive data capture and the utility of COVID-19 related questionnaire data.

## Background

The emergence of the COVID-19 pandemic led to many new data collection initiatives and the expansion of existing ones in order to assess the impact of the pandemic on certain populations [1]. Common ways to do this were the inclusion of imported Electronic Health Records (EHRs), the development of participant completed questionnaires, and the conducting of lab tests on provided biospecimens. Aspects being studied often included vaccine perceptions and usage [2, 3], infection severity [4, 5], common exposures [6], reactions to changing policies [7, 8], and overall lifestyle changes [9]. Along with the initial developed framework of COVID-19 data capture initiatives, many changes to the type and breadth of data being collected changed over time to understand changes in the pandemic, as well as individuals’ exposures and perceptions.

One such program that expanded their data collection to assess these factors during the COVID-19 pandemic was the UK Biobank program. UK Biobank is a large-scale research and biomedical data collection resource that supports multiple retrospective observational studies [10–12]. The program includes several types of physical measurement, questionnaire, diagnosis, procedure and genetic data for a population of individuals in the United Kingdom (UK) [13]. The data is constantly enriched with new data fields and new data for existing fields to aid in developing research topics.

Over the COVID-19 pandemic, UK Biobank included many new data fields relating to the pandemic. This included a COVID-19 exposure questionnaire and a COVID-19 symptom questionnaire (which was conducted twice). This data was part of the UK Biobank serological studies that also included serological tests to understand immunity over time for the UK Biobank participants. The official names of the questionnaires were the waves 1–6 exposure questionnaire, the waves 1–6 symptom questionnaire and the wave 7 symptom questionnaire. The ‘waves’ labels on the questionnaires pertain to the waves of the serological study and have no connection to waves of the COVID-19 pandemic itself. For each questionnaire, waves 1–6 and wave 7 are referred to as W1-6 and W7 respectively. Additional data in the UK Biobank dataset also included imported COVID-19 viral test data and hospital inpatient diagnostic data, which help provide context and lead to a better assessment of the complete effect of the COVID-19 pandemic [14].

This study analyzed the COVID-19 data included in the UK Biobank program, with the goal of understanding how the program was expanded to include COVID-19 data and how it can be used to attribute COVID-19 and analyze differences in cohorts and time periods as it relates to the pandemic.

## Methods

### COVID-19 cohort

To determine if an individual had a COVID-19 infection, three different methods were used including three different types of data. The first was via a self-reported positive lab test that was part of the provided questionnaire in the base data file. The second was COVID-19 viral test data provided through the data portal [14]. The third was by using diagnostic data that included hospital inpatient data imported from EHRs provided in the data portal and in the base dataset [15]. The difference in participant capture of these three methods was assessed and it was determined if there was any crossover or exclusion created by the different attribution methods. A single instance of any of these methods was deemed sufficient to consider an individual as having had a COVID-19 infection. A cohort of COVID-19 participants (referred to as the COVID cohort) was defined as any participant captured by one of the attribution methods. The non-COVID cohort was any other participant present and living at the beginning of the pandemic, who was not part of the COVID cohort and completed the studied questionnaires.

Over the course of the pandemic the dynamics and severity of the pandemic changed. To better understand this, changes in positive COVID-19 cases for the UK Biobank population over time was assessed as well as changes in the different attribution methods over time.

### UK Biobank COVID questionnaires

For a subset of the enrolled participants, UK Biobank collected a variety of voluntarily completed COVID-19 questionnaire data [16]. Each of the questionnaires completed by UK Biobank participants were analyzed. The questionnaires included the exposure questionnaire and two versions of the symptoms questionnaire (W1-6 and W7), which were given at two distinct times.

For the exposure questionnaire each question was analyzed by calculating the amount of times a question was answered, each response was chosen, and how many distinct participants that provided each response.

In the exposure questionnaire, UK Biobank designed a set of questions that asked about specific time periods to understand changes in the population’s exposure over time. For conciseness, these time periods are referred to by their numbers 1 to 4. Table 1 shows the dates for each time frame including W1-6 and W7 which pertain to the overall time frame for each questionnaire.

**Table 1:** Timeframes for each questionnaire

Time Period	Date Range
T1	mid-March through June 2020
T2	July through mid-September 2020
T3	mid-September through October 2020
T4	November through December 2020
W1-6	May through December 2020
W7	November 2021 through March 2022

Changes in the questionnaire dynamics were analyzed by calculating the number of participants who provided responses regarding each time period. To understand the importance of capturing different time frames, how often individuals changed their response from one time period to the next was calculated. Overall response breakdown for these different time periods was also analyzed to understand changes that occurred on a population-wide level.

While the previously mentioned questions with a time period stated does not allow for exact analysis of change in response around a COVID-19 diagnosis, it is possible to assess changes before and after COVID-19. Using the date of a COVID-19 diagnosis or positive lab test (self-reported did not include a date and were excluded), responses in the time period preceding the diagnosis, the time period during which the diagnosis occurred, and the time period after a COVID-19 infection when available were analyzed. These time periods are referred to as *before*, *during* and *after*. For each time period, how often an individual changed their response to the questions with a time component was analyzed across the different time periods. The populations response breakdown during each of these three time periods was also analyzed to assess any differences in exposures and lifestyles leading up to and after a COVID-19 infection as well as changes population-wide in the same way as defined before for the overall population.

Aside from the exposure questionnaire the UK Biobank COVID-19 data included a symptom questionnaire, which asked individuals whether or not they had a given symptom or required a certain level of medical attention. The symptom questionnaire was done twice, covering March through December 2020 (referred to as W1-6) and November 2021 through March 2022 (referred to as W7). Any changes in the questionnaires from the first version (W1-6) to the second version (W7) were reviewed as well as any changes in responses that occurred between the two questionnaires.

All analyses of the questionnaire data were done for the entire UK Biobank population who completed the questionnaires, the COVID cohort and the non-COVID cohort using a repeatable data science driven R script developed for the study [17].

### Comparison of COVID and non-COVID responses

It is well known that different lifestyles and exposures may make it more likely for an individual to be exposed to and infected with COVID-19 [6, 18–20]. With this in mind, the exposure questionnaire responses of the COVID cohort (individuals self-reporting, testing positive or diagnosed with COVID-19) were compared to those that were not part of the COVID cohort. This was possible as the repeated methodology used for each cohort gave comparable results with identical structured results. The COVID cohort for comparison of the exposure questionnaire was limited to those who had COVID-19 (based on the attribution methods outlined above) during the time the questionnaire was given (2020).

In addition to comparing results from the exposure questionnaire, the results of the symptoms questionnaire was also compared between the COVID and non-COVID cohorts. Based on the results of the symptoms questionnaire it is possible to identify individuals who likely had a COVID-19 infection but were undiagnosed and never tested positive. The reporting of significant symptoms for individuals not captured in the COVID cohort or the requirement of a certain level of care for these individuals may indicate that they have had a COVID-19 infection despite not being captured in the COVID cohort based on the used attribution methods.

### COVID-19 vaccination

To further assess population characteristics, UK Biobank asked their population if they received a COVID-19 vaccination. This included asking if and when a first and second vaccine were received. The proportion of individuals who received a first and second vaccine was calculated as it relates to the total number of participants who provided a vaccination status.

In addition, an assessment of the affect a COVID-19 infection had on receiving a vaccine was analyzed. This was done by calculating how many individuals and the percentage of the population that received a COVID-19 vaccine after having COVID-19 (as defined above in cohort identification) as well as how often an individual had a COVID-19 infection after they received the vaccine.

## Results

The complete set of results and supplementary materials can be found at the project repository CRI/UKBB/COVID_Questionnaire at master lhncbc/CRI (github.com).

### COVID attribution and cohort

Overall, UK Biobank includes 501 541 individuals at the time of analysis. At the beginning of the pandemic (as of March 2020) 472 431 were still living and able to accrue COVID-19 related data. There were 434 104 COVID-19 viral tests done for 168 351 (6.09%) UK Biobank participants in the provided testing data. 15 338 (50.20%) people testing positive for COVID-19 and 76 825 (55.75%) people who did not test positive for COVID-19 had only one viral test in the available data indicating more people who tested positive for COVID-19 had a repeat test while those testing negative commonly did not have an additional COVID-19 test. Table 2 shows the distribution of the COVID cohort by attribution method including the count of individuals with data in the fields required for that attribution method. The participant counts include individuals that crossover between multiple attribution methods.

**Table 2:** COVID-19 identification by domain

	Number of participants with COVID-19	Number of participants present in data field	Percentage of present in data field	Percentage of total UK Biobank participants
Lab Measurement	30 553	168 351	18.15%	6.09%
Diagnosis	49 120	447 281	10.98%	9.79%
Self-reported	934	10 890	8.58%	0.19%

In total, across the different methods, there were 62 042 distinct participants who had a COVID-19 infection based on one or multiple of these methods. Of the 934 participants self-reporting a positive test 133 were in the first (W1-6) symptom questionnaire and 780 in the second (W7). Of the 49 120 individuals who had a listed diagnosis code for COVID-19, 31 064 (63.24%) did not have a positive viral test in the provided testing data. 18 056 participants were identified as having COVID-19 via both the diagnosis and measurement data, while 735 were in both the self-reported and diagnosis, and 453 were captured in both the self-reported and measurement data. In total 61 617 (99.32%) participants were identified by a medical professional (either a diagnosis or positive test, excluding those only identified via self-reporting) as having COIVD-19.

**Fig. 1:**
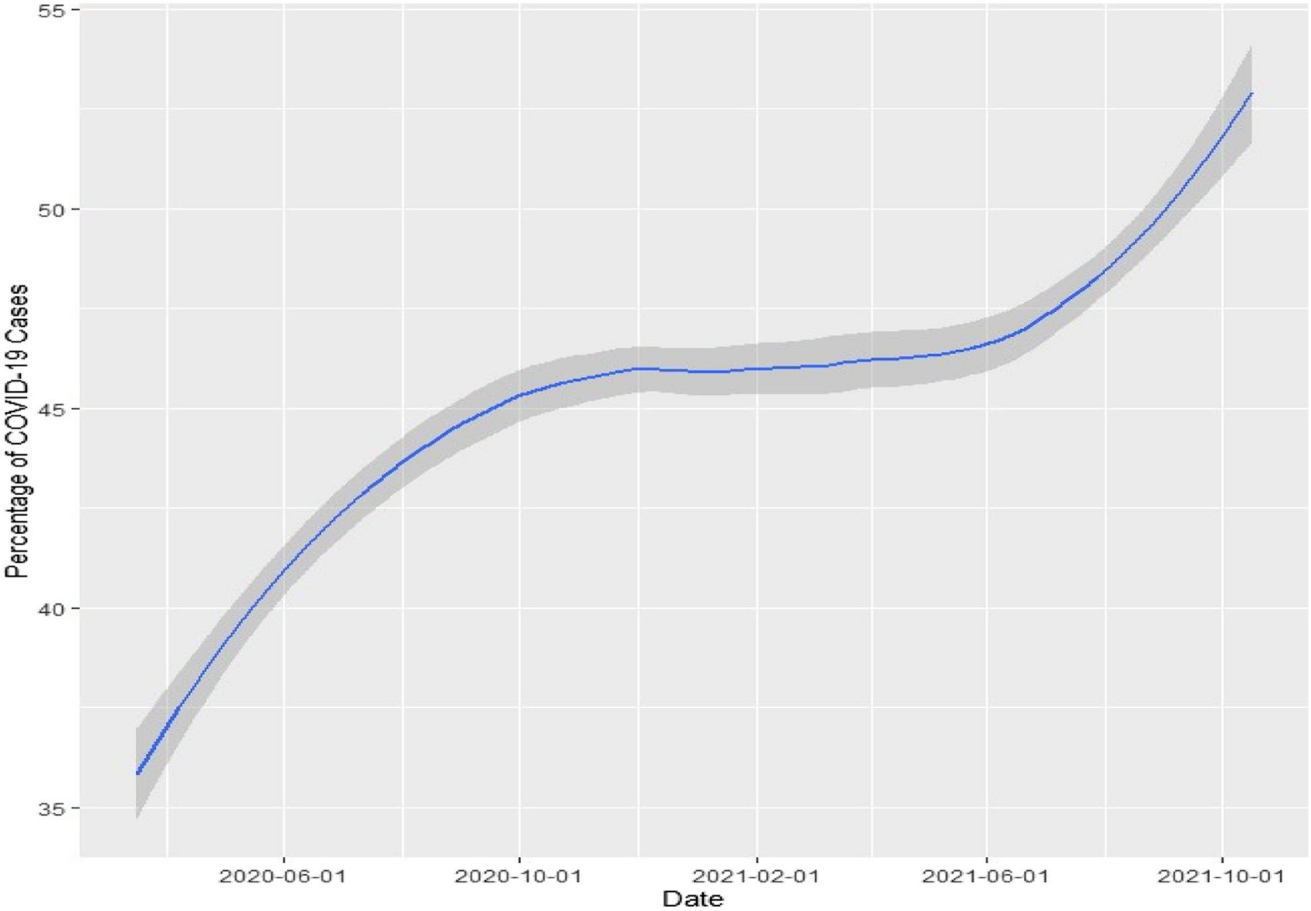
Percentage of COVID-19 cases identified from testing data by date

The amount of testing available changed drastically over the course of the pandemic. Figure 1 shows the general trend for the percentage of participants with COVID-19 identified from the available testing data as a percentage of all identified COVID-19 participants on a given date.

As shown by Fig. 1, the percentage of individuals identified via testing data (as a percentage of total cases at a time) increased from the beginning of the pandemic as more testing became available. Also of note is that 490 participants were diagnosed with COVID-19 before the available testing data (prior to March 2020).

On a demographic level, the average age of the individuals with a COVID-19 infection was 66.39, with 34 074 (54.92%) being Female. In comparison the average age of those without a COVID-19 infection was 68.92 and 54.22% female.

#### COVID-19 cases over time

**Fig. 2:**
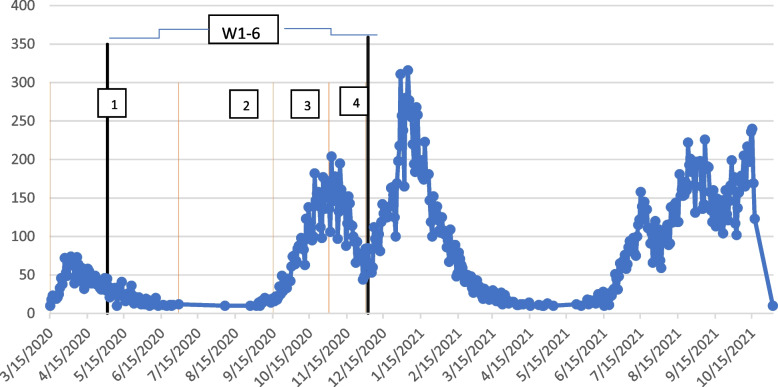
COVID-19 cases over time for the UK Biobank population

There were many changes in the dynamics over the course of the pandemic with many increases and decreases in COVID-19 cases over time. Figure 2 shows the number of COVID-19 cases for the UK Biobank population over time, as well as the time periods represented by the different questionaries.

Figure 2 shows the cases during the W1-6 timeframe (denoted by bolded black lines) and the different time periods included in time specific questions (denoted by the orange lines and numbered regions). As can be seen by Fig. 2, time period 1 includes the initial wave of the pandemic. Time period 2 contains a lull in COVID-19 cases after the initial lockdown in the UK, while time periods 3 and 4 show the growing and waning of a new wave of cases respectively. As can also be seen from Fig. 2 there are multiple waves outside these time periods. W7 is not on the figure as it occurs after the available COVID-19 infection data (ending October 2021). Table 3 shows the total number of cases in these time periods.

**Table 3:** COVID-19 cases by time frame

Time Period	COVID-19 Cases
T1 (mid-March through June 2020)	1 873
T2 (July through mid-September 2020)	187
T3 (mid-September through October 2020)	3 536
T4 (November through December 2020)	2 838
W1-6 (May through December 2020)	6 965

### COVID questionnaire populations

The number of participants completing each questionnaire varied. The exposure questionnaire was completed by 9 952 (2.11%) participants, the W1-6 symptoms questionnaire was completed by 10 878 (2.30%) participants and the W7 symptom questionnaire was completed by 8 445 (1.79%) participants. 8 433 participants completed both symptom questionaries, while 2 445 only completed the first (W1-6) symptom questionnaire and 12 only completed the second (W7) one.

For the analysis of the exposure questionnaire the COVID cohort consisted of 11 980 (19.31%) participants who had COVID-19 (based on the previously outlined attribution method) during the time the questionnaire was referring to (at any point between March and December 2020). Of those, 314 (2.62%) completed the exposure questionnaire. Of all individuals attributed with COVID-19, 1 070 completed the W1-6 symptoms questionnaire, and 886 completed the W7 symptoms questionnaire.

#### Questionnaire dynamics

The exposure questionnaire consisted of 94 questions with 20 questions answered by all 9 952 individuals participating in the questionnaire. Table 4 shows the results for a subset of questions from the exposure questionnaire for the total population. For all the results, please visit the project repository [17].

**Table 4:** COVID exposure survey results for the whole UK Biobank population who completed the questionnaire

Question	Answer	Participant Count	Participant Percentage
Description of main home	A mobile home or caravan	20	0.20%
	An apartment or flat with no outside space	511	5.13%
	An apartment or flat with outside space	1 012	10.17%
	A house or bungalow	8 379	84.19%
Lives with others in main home	No	2 521	25.34%
	Yes	7 412	74.51%
Time spent per day within 1 m of people with COVID-19	Less than 10 min	81	22.44%
	More than 2 h	203	56.23%
Employment situation between mid-March and the end of June 2020 (i.e. the first UK lockdown)	Employed/Self-employed and working with others at a workplace	1 393	14.00%
	Employed/Self-employed and working from home	1 987	19.97%
	Retired	5 364	53.90%
In close contact with public or co-workers between 1st March 2020 and 30th November 2020 as part of job	Yes	1 716	17.24%
	No	6 841	68.74%
How often cloth face coverings used at work between 1st March 2020 and 30th November 2020	Half of the time	53	4.68%
	Most of the time	209	18.45%
	All of the time	238	21.01%
	Some of the time	268	23.65%
	Never	365	32.22%

Meanwhile, the symptom questionnaire was given twice (W1-6 and W7). Both versions of the symptom questionnaire, consisted of 16 questions regarding specific symptoms and three regarding the type of care required. One exclusion from the second version was a question regarding when symptoms began. This question was present on the first (W1-6) symptom questionnaire and not the second (W7), making it impossible to determine when the symptoms declared on the second questionnaire occurred in relation to any other factors. The symptom questionnaires also included the self-reported positive tests previously used in COVID-19 attribution. Table 5 shows the participant counts and percentages for symptoms in W1-6, W7 and combined for the complete UK Biobank population. The combined count in Table 5 may include individuals who reported symptoms in both W1-6 and W7 but are only counted once in the combined count. For example, 1 281 participants reported feeling more tired than usual on both W1-6 and W7 which is why there is a combined number different from what would be found by adding W1-6 and W7 counts.

**Table 5:** Symptom participant counts for the entire UK Biobank population who completed the questionnaire

Question	W1-6 Count	W1-6 Percentage	W7 Count	W7 Percentage	Combined Count	Overall Percentage
Feeling more tired than usual	3627	33.34%	2258	26.74%	4604	42.28%
Runny nose	3147	28.93%	2549	30.18%	4460	40.96%
Headache	3370	30.98%	1958	23.19%	4153	38.14%
Sore throat	3086	28.37%	1717	20.33%	3911	35.91%
Muscle ache	2496	22.95%	1568	18.57%	3319	30.48%
COVID-19 symptoms requiring medical attention	1567	14.41%	1376	16.29%	2607	23.94%
Diarrhea	1863	17.13%	763	9.03%	2239	20.56%
Persistent dry cough	1605	14.75%	773	9.15%	2138	19.63%
Shortness of breath	1544	14.19%	756	8.95%	1968	18.07%
Chills	1247	11.46%	783	9.27%	1779	16.34%
COVID-19 symptoms requiring self-isolation	1182	10.87%	698	8.27%	1769	16.24%
Productive long-term cough ('wet' or chesty)	1100	10.11%	682	8.08%	1566	14.38%
COVID-19 symptoms: Abdominal pain	1150	10.57%	495	5.86%	1450	13.31%
Fever 38 degrees C or greater	957	8.80%	303	3.59%	1195	10.97%
Wheezing	878	8.07%	475	5.62%	1182	10.85%
Nausea/vomiting	888	8.16%	377	4.46%	1157	10.62%
Loss of sense of smell and taste	768	7.06%	448	5.30%	1115	10.24%
Chest pain	766	7.04%	342	4.05%	989	9.08%
COVID-19 symptoms requiring hospitalisation	111	1.02%	43	0.51%	153	1.40%

Table 5 shows that only one symptom, runny nose, increased in patient percentage from the first to the second questionnaire. All other symptoms decreased. Symptoms requiring medical attention also increased in frequency from the first version to the second, but symptoms requiring hospitalisation decreased.

#### Changes over time

On the exposure questionnaire, there were 14 questions that included a time component. The individuals answering each question varied as 7 831 answered regarding T1, 8 612 about T2, 8 422 about T3, and 8 050 about T4. Overall, 6 899 (75.04% of participants responding to the questionnaire) provided responses for all time periods and could be followed through the course of the questionnaire timelines. The other 2 295 (24.96%) participants did not respond to questions regarding every time period and instead only answered questions regarding a portion of the time periods asked about.

To understand changes caused by the time periods two types of changes were assessed, participant, and population levels. On a participant level, Table 6 shows the proportion of people who changed their responses from the previous time period for each question.

**Table 6:** Response changes across different time periods

Question	Response change from T1 to T2	Response change from T2 to T3	Response change from T3 to T4
Days per week meeting friends and family at an inside venue	50.49%	35.22%	37.20%
Days per week meeting friends and family at an outside venue	47.44%	34.85%	40.09%
Employment situation	58.95%	24.67%	19.98%
How often alcohol-based sanitiser used when outside home	22.51%	14.04%	13.00%
How often disposable gloves worn when outside home	14.80%	8.03%	6.49%
How often eye protection used when outside home	4.01%	3.04%	2.84%
How often face mask used when outside home	36.87%	23.75%	20.27%
Left home for reason other than paid work	15.39%	8.68%	9.26%
Left home to eat or drink inside a cafe, restaurant or pub/bar	51.51%	30.35%	34.08%
Left home to go shopping	27.92%	18.40%	18.98%
Left home to visit a healthcare setting	28.91%	21.73%	25.11%
Main mode of transport	23.45%	12.92%	12.73%
Time in close contact with people	44.82%	29.22%	28.85%
Young people in main home attended school or other childcare setting	35.29%	30.49%	7.84%

From Table 6 it can be seen that for each question the most common response changes came in the change from T1 (mid-March through June 2020) to T2 (July through mid-September 2020). It is of note that within questions regarding T1, UK Biobank includes a statement calling the time period ‘the first UK lockdown’. This implies the loosening of policies in T2 that were put in place in T1, which would contribute to the changes in responses between the two time periods. For three of these questions more than 50% of respondents changed their answer from the first time period to the second where the question with the most respondents changing their answer being Employment situation (58.95%). For Employment Situation the affect lasted through each succeeding time period as 74.78% of respondents changed their response from the first time period to the last, the most of any question.

**Fig. 3:**
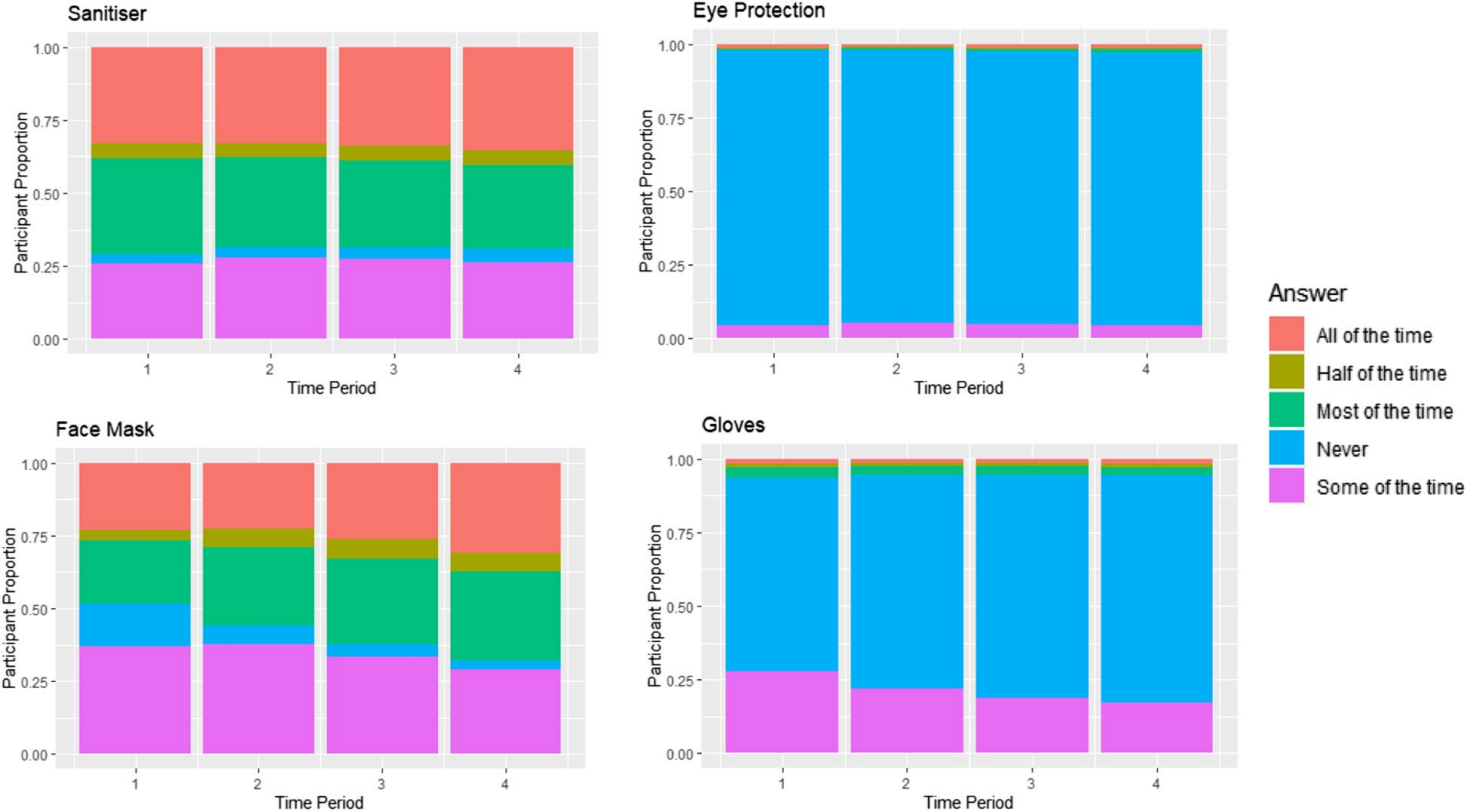
Proportions of participants taking certain safety precautions during each time period

**Fig. 4:**
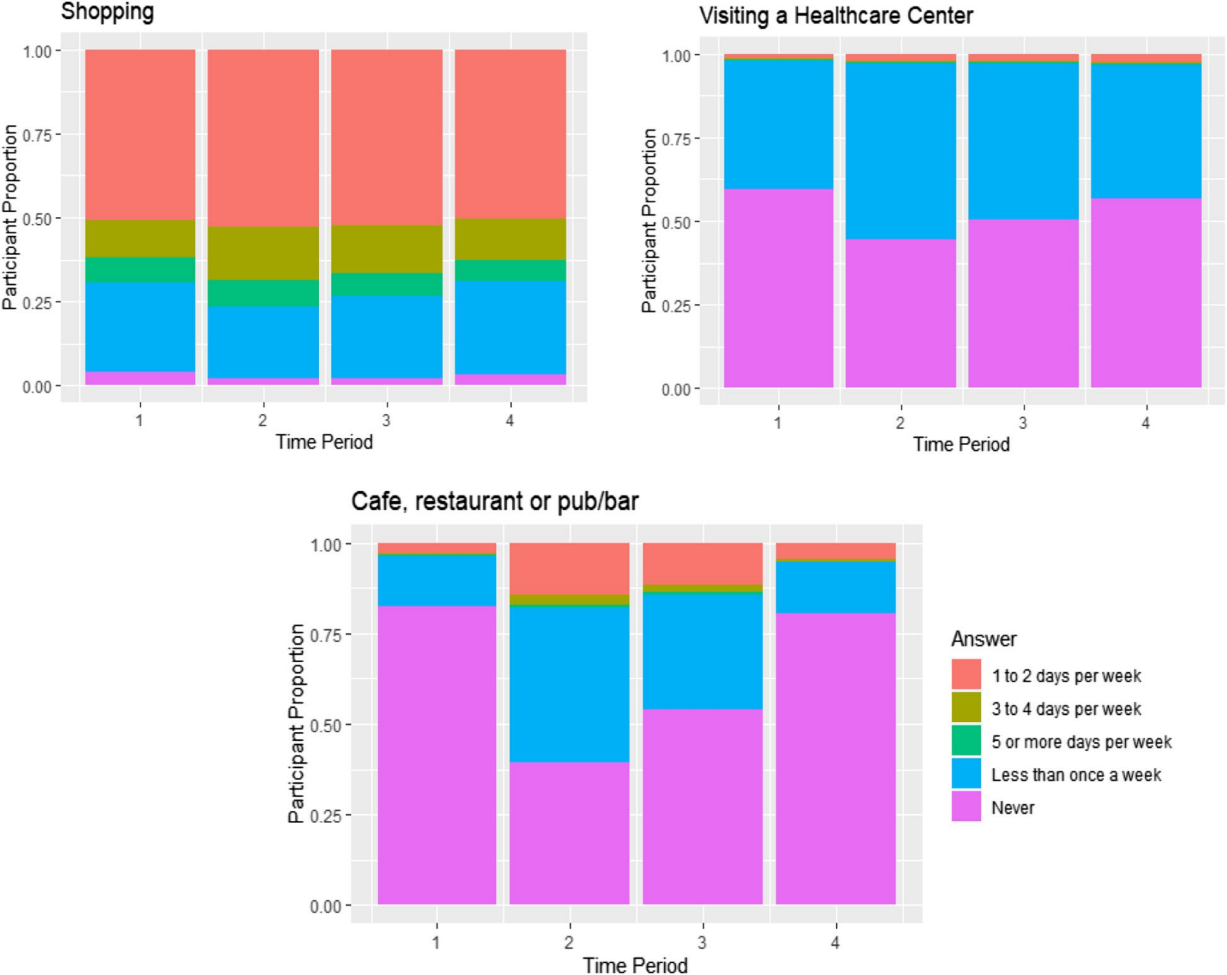
Patient proportions by reason for leaving home during each time period

When looking at the population level breakdown, Figs. 3 and 4 show the proportion of participants who gave certain answers to a given question.

Figure 3 shows responses to questions regarding safety precautions relating to eye protection, face masks, gloves, and sanitisers. As can be seen by Fig. 3 the use of face masks increased over time during 2020 as more people changed their response from ‘never’ and ‘some of the time’, to ‘most of the time’ or ‘all the time’. A similar trend, to a lesser extent, was seen for the use of hand sanitiser starting in the second time period. The use of eye protection and gloves were never as prevalent as the other safety precautions during any time period, although those that used gloves ‘some of the time’ decreased over time as they changed their answer to ‘never’.

Figure 4 shows the responses to multiple questions regarding frequency of leaving home for varying reasons. Overall going to cafes, restaurants and pubs/bars became more frequent from July through October 2020 (after the end of ‘the first UK lockdown’) and then was greatly reduced again in November and December 2020. Shopping became a more frequent occurrence after the initial time period (mid-March through June 2020), while visiting a healthcare setting was never really common but became more frequent in the middle time periods (July through October 2020).

#### Responses before, during and after COVID-19 infection

While the time specific questions in the exposure questionnaire refer to time periods it is possible to assess changes based on a COVID-19 infection by comparing the previous (before), current (during) and post (after) time periods around the time of the COVID-19 infection (based on the attribution methods outlined above). 3 832 participants were infected at a point where questions referring to before and after are present. 10 122 were infected at a time when before and during was reflected in the questions while 5 998 were infected at a time where during and after were observable. Table 7 shows the percentage of participants who changed their response around their COVID-19 infection.

**Table 7:** Response changes over time by COVID participant

Question	Before to During	During to After	Before to After
Left home to eat or drink inside a cafe, restaurant or pub/bar	25.36%	15.08%	27.81%
How often face mask used when outside home	22.37%	13.03%	27.32%
Days per week meeting friends and family at an outside venue	21.27%	12.10%	22.90%
How often disposable gloves worn when outside home	15.36%	6.77%	16.67%
How often alcohol-based sanitiser used when outside home	15.09%	10.03%	19.13%
Days per week meeting friends and family at an inside venue	14.89%	16.00%	21.74%
Young people in main home attended school or other childcare setting	13.21%	7.52%	14.75%
Left home for reason other than paid work	12.67%	8.02%	15.03%
Employment situation	10.84%	5.96%	11.66%
Left home to go shopping	8.89%	5.26%	8.74%
How often eye protection used when outside home	6.25%	5.19%	6.25%
Main mode of transport	5.93%	3.26%	7.10%
Time in close contact with people	4.04%	3.51%	4.92%
Left home to visit a healthcare setting	1.08%	1.26%	1.09%

For 12 questions a higher percentage of individuals changed their response from before to during as compared to those who changed their response from during to after infection.

**Fig. 5:**
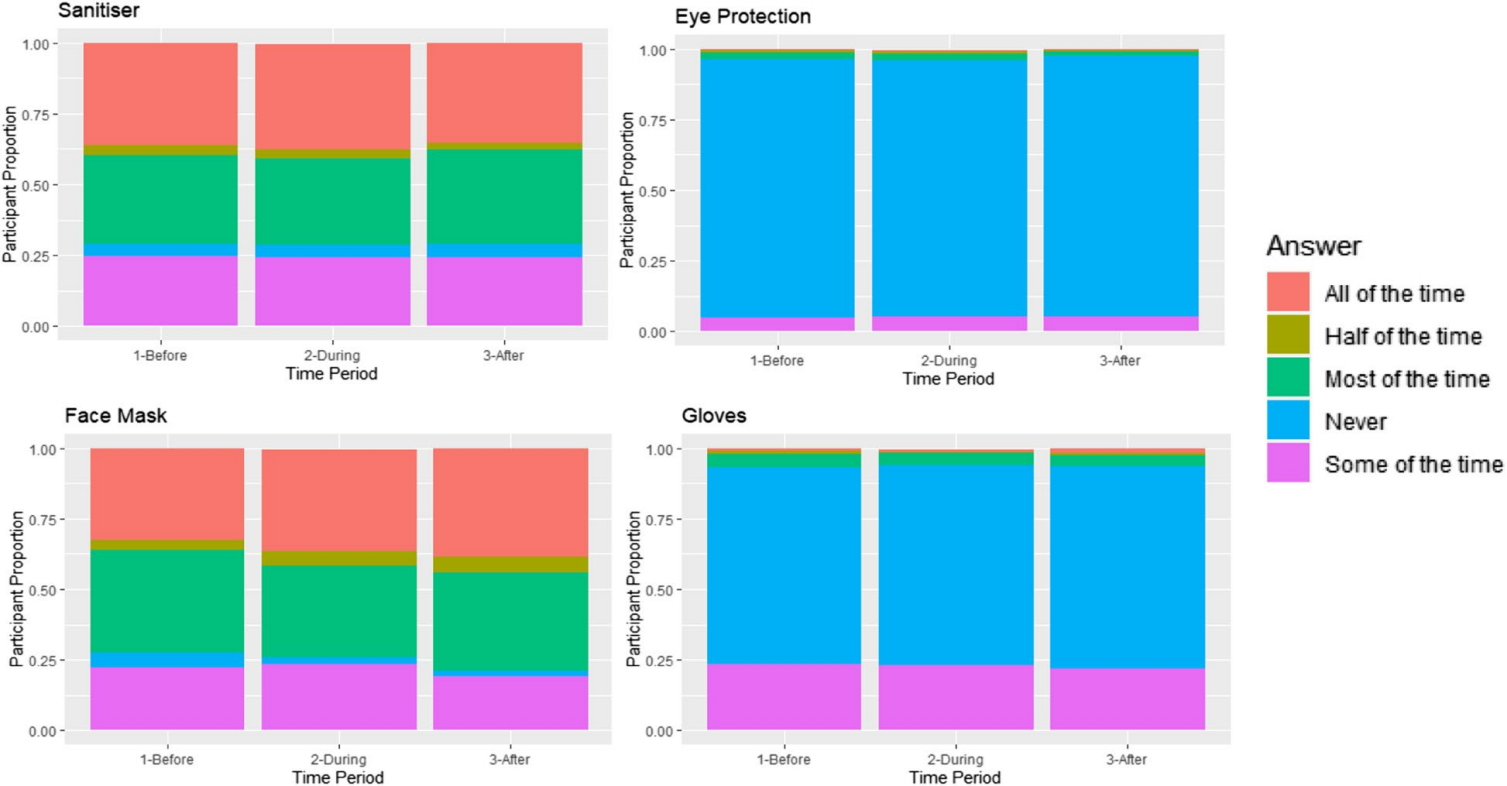
Participant proportions for safety precautions before, during, and after COVID

Using time around a COVID-19 infection, changes in lifestyle were discovered in time periods surrounding a COVID-19 infection. Figure 5 shows trends on a population level for the COVID cohort in the time periods before, during and after a COVID-19 infection for safety precautions taken.

As can be seen in Fig. 5, face mask usage increased from before to after infection, while the use of hand sanitiser ‘all of the time’ increased during the time period when the COVID-19 infection occurred as compared to before and after.

### Comparison of COVID cohort to non-COVID population

Based on percentage of respondents choosing a specific answer to a question, common differences between the non-COVID and COVID cohort can be seen in Table 8. The full comparison of the COVID and non-COVID populations can be seen at the project repository.

**Table 8:** Comparison of COVID and non-COVID exposure questionnaire responses

Question	Answer	COVID Percentage	Non-COVID Percentage	Difference	P-Value
Young people in main home attended school or other childcare setting between the start of July and mid-September	Yes	61.70%	45.32%	16.38%	0.011
	No	36.17%	52.52%	-16.35%	0.010
Number of people in main home tested positive for COVID-19	1	62.94%	28.69%	34.25%	0.032
	2	15.38%	2.62%	12.76%	0.065
	0	13.29%	64.28%	-50.99%	0.029
Employment situation between mid-March and the end of June 2020 (i.e. the first UK lockdown)	Employed/Self-employed and working with others at a workplace	31.21%	13.44%	17.77%	0.012
	Employed/Self-employed and working alone at a workplace	3.82%	2.45%	1.37%	0.005
	Employed/Self-employed and working from home	19.11%	19.99%	-0.89%	0.001
	Retired	36.31%	54.47%	-18.17%	0.003
Designated key worker between 1st March 2020 and 30th November 2020	Yes	33.76%	15.00%	18.75%	0.012
	No	60.83%	77.56%	-16.73%	0.002

Overall, 1 070 individuals who self-reported, tested positive or were diagnosed with COVID-19 completed the first (W1-6) symptom questionnaire compared to 886 who completed the second (W7). Although the second questionnaire was after the end of the available COVID-19 infection data it is reasonable to believe that the symptoms denoted in the questionnaire by individuals who had COVID-19 pertain to their infection and are either symptoms from when the infection occurred or persisted after the initial infection. Table 9 shows the counts and proportion of participants with specific symptoms for each version of the questionnaire for the COVID cohort.

**Table 9:** Symptom participant counts for the COVID population

Symptoms	W1-6 Count	W1-6 Percentage	W7 Count	W7 Percentage	Percent Change W1-6 to W7
Feeling more tired than usual	450	42.06%	548	61.85%	-19.79%
Headache	410	38.32%	408	46.05%	-7.73%
Runny nose	373	34.86%	394	44.47%	-9.61%
Sore throat	368	34.39%	335	37.81%	-3.42%
Muscle ache	317	29.63%	390	44.02%	-14.39%
Diarrhea	242	22.62%	134	15.12%	7.49%
Persistent dry cough	206	19.25%	253	28.56%	-9.30%
Shortness of breath	204	19.07%	251	28.33%	-9.26%
Chills	188	17.57%	252	28.44%	-10.87%
Abdominal pain	150	14.02%	89	10.05%	3.97%
Productive long-term cough ('wet' or chesty)	145	13.55%	161	18.17%	-4.62%
Fever 38 degrees C or greater	141	13.18%	162	18.28%	-5.11%
Wheezing	125	11.68%	159	17.95%	-6.26%
Nausea/vomiting	124	11.59%	87	9.82%	1.77%
Loss of sense of smell and taste	117	10.93%	265	29.91%	-18.98%
Chest pain	110	10.28%	109	12.30%	-2.02%

Overall, in the first (W1-6) questionnaire, 22.99% (246 participants) of COVID participants had symptoms requiring medical attention, while 1.68% (18 participants) had symptoms requiring hospitalisation. This compares to 46.84% (415 participants) from the second (W7) questionnaire requiring medical attention and 2.37% (21 participants) requiring hospitalisation. On average in the first questionnaire each individual with COVID-19 reported 5.5 symptoms compared to an average of 6.8 symptoms per person in the second questionnaire. 7 073 participants reported symptoms but did not have a captured COVID-19 infection. The most reported symptoms by those with no stated COVID-19 infection were more tired (3 871 participants, 54.73% of those reporting symptoms with no COVID-19 infection) and runny nose (3 857 participants, 4.53%). Of note 2 034 required medical attention and 114 required hospitalisations for reported symptoms but had no COVID-19 diagnosis or positive viral test captured and did not self-report a COVID-19 infection in the provided data.

### COVID-19 vaccination

Table 10 shows a summary of metrics regarding the vaccination status of the UK Biobank participants. As can be seen from Table 10 only a subset of total participants (201 890) provided their vaccination status. 195 400 (96.48% of people who provided vaccination status) via the questionnaire data said they received at least one COVID-19 vaccination. 29.64% of those who received the first dose also received a second. No indications of COVID-19 vaccination were detectable in either of the drug or procedure data provided. There was also no declaration of which COVID-19 vaccination was received by different individuals making it unclear how many doses of the vaccine were required as part of the initial regimen.

**Table 10:** Summary of counts by vaccination sattus

Metric	Participant Count
Provided Vaccination Status	201 890
Received One Vaccination	117 936
Received Two Vaccinations	77 464
Unvaccinated and had COVID-19	6 463
Had COVID-19 and provided vaccination status	32,413
Vaccinated and Had COVID-19	30 108
Unvaccinated and had COVID-19	2 305
Vaccinated and did not have COVID-19	184 002
Unaccinated and did not have COVID-19	6 274

Table 10 also shows the count by vaccination status of the COVID cohort. 14 184 (47.11% of 30 108) received the vaccine at some point after they had COVID-19 compared to 15 924 (52.89%) who received the vaccination prior to having COVID-19.

## Discussion

### Cohort validation

While UK Biobank included a data field for self-reporting positive COVID-19 tests, it was not sufficient to capture all participants who had COVID-19. Only 934 self-reported a positive COVID-19 test. Once taking into account the listed hospital and inpatient diagnosis codes (in the base data set and data portal) and testing data (in the data portal) the number of COVID-19 cases rose 66 times (to 62 042 participants). This showcases the need for multiple sources for COVID-19 case identification when observing the complete time period of the pandemic due to the fluctuation in viral test availability and reporting. Even the inclusion of just viral test data would not have been sufficient as 31 489 (50.75%) COVID-19 diagnosed participants would have been excluded if self-reported and diagnosis data were not included. This is likely caused by either the fact testing was not available at the time of diagnosis (especially early in the pandemic as shown in Fig. 1) or the emergence of home self-tests. Of those self-reporting a positive test, but with no positive test in the supplied viral testing data, 76.00% reported the positive test as occurring after 2020, corresponding with the increase usage of home self-tests [21, 22]. This capture of distinct individuals for cohort identification from the different methods showcases the need for inclusion and usage of multiple methods of attributing medical conditions in cohort identification within a single dataset.

The increased possibility of the use of self-tests and other methods that may not have been captured in the provided data leaves the possibility that not all COVID-19 infected participants were captured in the COVID cohort. Also due to the nature of the pandemic, the commonness of being asymptomatic and the availability and accessibility of testing early on in the pandemic it is likely participants had COVID-19 but were not tested or diagnosed and thus not captured in the data. By assessing the differences in responses to the exposure and symptom questionnaires, it is plausible to determine possible undiagnosed COVID-19 participants. Individuals with responses that more closely resemble the COVID cohort’s responses, could possibly be undiagnosed COVID-19 individuals. For example, if an individual stated they lived with someone who had COVID-19 (COVID cohort: 86.71%, non-COVID cohort: 35.72%) or worked in a workplace with others (COVID: 31.21%, non-COVID: 13.44%) they are more likely to have had an undiagnosed COVID-19 infection then someone who answered the questions differently. In addition, if an individual had symptoms requiring medical attention or hospitalisation but did not have a listed COVID-19 diagnosis, they may have had COVID-19 and were not diagnosed as seen by the level of care. This is also increasingly possible with the fact that the second symptom questionnaire was given after the end of data collection used to attribute COVID-19 to individuals.

Since only a single instance and source was used for inclusion in the COVID cohort (one positive lab test, one diagnosis code or one self-reported positive lab test) it is likely that some individuals included in the cohort were misdiagnosed or miscategorized in the analysis. Due to the availability and reliability of testing at various points in the pandemic, misdiagnosed cases of COVID-19 is a common problem in cohort determination [23, 24], It is possible to identify such individuals in the cohort by reviewing their responses similar to finding undiagnosed individuals previously discussed. Individuals with no symptoms and no confirmatory result may have been misdiagnosed or had a false positive test, though further study of such possibility would be needed.

Another consideration is the differences in the type of sample and lab test used, which was not differentiated in the analysis. While it is not known what type of lab test was used, there were 53 distinct types of specimens used in the lab testing data. This variability could affect the validity of the test result [25]. Using the previously discussed questionnaire responses it may be possible to identify individuals who were miscategorized.

While the study did not differentiate between one continuous infection compared to reinfection in the time analysis (since they were related to time periods and not a specific date), it is feasible to differentiate if necessary. Such differentiation was shown by Dong et al. [26].

### Vaccination attribution

To determine vaccination status for the participants questionnaire data had to be used. Despite UK Biobank including procedure and drug information, no information regarding the COVID-19 vaccine was present in either. Without the inclusion of COVID-19 vaccinations in imported EHR data, as either a drug or procedure, it was not possible to validate the responses to vaccination status. Including this information would allow for the ability to track if any individuals received a vaccination or additional doses after the questionnaire was given and identify differences in fully and partially vaccinated individuals based on the amount of COVID-19 vaccines received over time. Despite this missing information the total number of identified individuals and percentage of the population aligns with the numbers reported by NHS for the same time frame. The NHS data states 95.31% of the older population had at least one vaccination by June 2021 [27]. This lines up with 96.48% of the studied population for the same time frame.

### Changes over time

Many of the observed time trends in responses can be linked to changes in case counts, as in many cases changes in COVID-19 cases affected individual lifestyles, as well as the reverse, that changes in lifestyles showed changes in case count trends. These changes may have been impacted by policies implemented in the different time periods. For instance decreases in leaving home for various reasons in November and December 2020 can be traced to restrictions implemented during that time period [28]. Similar policy affects can be seen in healthcare utilization trends based on the implementation and relaxing of lockdowns during the early parts of the pandemic [29]. This aligns with the previously stated results regarding frequency of visiting a healthcare setting for the first and second time period studied.

The inclusion of multiple timeframes and the fact that 75.04% of participants completing the exposure questionnaire provided responses regarding each timeframe allowed for the following of individuals changes in lifestyles over the course of the pandemic that would not have been possible if a single question spanning the whole pandemic was included instead. This is also true regarding 3 832 COVID-19 infected participants whose exposures could be tracked before and after the time of the COVID-19 infection.

While the exposure questionnaire captured multiple timeframes throughout 2020, the inclusion of additional timeframes in 2021 may have shown further changes to exposures. While the results showed many changes from the beginning of the pandemic through each subsequent time period, it was also notable that many of the changes from the first time period maintained through the last time period such as employment situation. The inclusion of further time periods may have showed the maintaining of these changes or a reversion back to the original responses from the initial time period, as changes in the severity of the pandemic continued after the end of the exposure questionnaire as was shown in Fig. 2.

There was substantial time between the first (W1-6) symptom questionnaire (March to December 2020) and the second (W7, November 2021 to March 2022). For the UK Biobank population as a whole, there was a notable decrease in nearly every symptom, except for runny nose) as well as a decrease in symptoms requiring hospitalisation. One explanation for this, was that during the time period between both questionnaires multiple COVID-19 vaccines were approved in the UK [30], which considerably lower the risk of symptoms and hospitalisations. This is increasingly clear as 96.48% of respondents said they received at least one COVID-19 vaccine, which may have reduced the risk of showing symptoms captured in the second symptom questionnaire as compared to the first. Conversely, however, was the fact that many symptoms actually increased for the COVID cohort from the first to the second symptom questionnaire. A couple of explanations for this is the possibility of long COVID symptoms being reported or symptoms that are referenced to the time prior to receiving the COVID vaccine as 14 184 participants had COVID-19 before receiving the vaccination.

### Common data elements

Common Data Elements (CDEs) are data elements that are present in multiple data collection [31, 32] sources and are useful for quick and easy comparative analyses across different data sources [31, 32]. While UK Biobank does not formalize any of their COVID-19 data into a standardized terminology there are efforts to transform the base data into the Observational Medical Outcomes Partnership (OMOP) common data model [33].

Manual review of multiple studies can lead to the discovery of CDEs. While UK Biobank is limited to UK participants, the use of CDEs allows for the comparison of the results to different nations and contexts. For example, the All of Us program is a large-scale research program in the United States that also added COVID-19 data including survey questions to assess the pandemic’s impact on the participants [34]. One such CDE present in both UK Biobank and All of Us is in regard to working status. In this case, 19.97% of the UK Biobank respondents answered they worked from home which compares to 32.15% of All of Us respondents.

### Related studies

While this study analyzed the comparison of exposures and factors associated with a COVID-19 diagnoses, there are many other studies analyzing various facets of the COVID-19 pandemic. Klann et al. studied the determination of COVID-19 severity phenotypes from EHRs across multiple sites along with site variability [35]. Such differences in severity of studied participants could affect questionnaire responses if stratified by location. Lin et al. also analyzed the problem of survey bias for mental health surveys during the COVID pandemic which can be a concern with other types of survey questions within the context of the COVID-19 pandemic [36].

### Limitations

The study has multiple limitations. First, a single diagnosis or positive test was relied upon to determine cohort inclusion and no attempt was made to confirm the accuracy of the cohort. This was necessary as less than half of the participants had multiple viral tests. This was slightly mitigated by the fact that those testing positive for COVID-19 more often had multiple viral tests when compared to those who did not test positive for COVID-19. There was also no attempt to identify misdiagnosed or undiagnosed individuals. Although geography may have an effect on participants responses it was decided to not stratify by location and did not analyze England, Wales and Scotland separately, although the lifestyle and pandemic trends may be different between the geographic locations [37, 38]. Stratification by location would have resulted in small sample sizes that may not have been sufficient for analysis. For the time analysis no determination of when a diagnosis occurred within a time period was done, just which time period it occurred in. When an individual had COVID-19 within a time period (at the beginning or end) may have affected whether they would have changed their response from one time period to the next.

## Conclusion

Analyzing UK Biobank data, it was observed that to develop the most inclusive cohort of COVID-19 infected individuals among UK Biobank participants, multiple identification methods and different data types were needed as 30 527 distinct participants came from provided viral test data, 31 064 from diagnostic data and 934 from self-reported data. This was also depicted by changes over time shown through increases in available viral tests and cases captured through testing over time. The dynamics of the questionnaires allowed for the tracking of individuals over time as well as changes in population level exposures over time. This fact showed many changes in safety precautions taken, outside activities and employment status and locations. The fact that a symptom questionnaire was given twice (at different times) also allowed for the finding of a decrease in most symptoms from the first to the second questionnaire that may have been affected by the widespread uptake of COVID-19 vaccines occurring between the two questionnaires. This was shown by the fact that a vast majority of participants reported receiving the COVID-19 vaccine. However, a lower percentage of individuals who had COVID-19 received a COVID-19 vaccination (92.89%) than those who did not have COVID-19 (96.70%). The analysis was also able to be repeated for different cohorts and many differences in exposures between individuals who had COVID-19 and those who did not were able to be identified. The use of COVID-19 related questionnaire data can provide valuable knowledge to lifestyles and exposures that impact COVID-19 infection and severity and how these factors changed over time during the course of the pandemic further showcasing the importance of comprehensive data capture and questionnaire structure and implementation.

## Data Availability

The data that support the findings of this study are available from UK Biobank but restrictions apply to the availability of these data, which were used under license for the current study, and so are not publicly available.
